# Contemporary management of high-grade gliomas

**DOI:** 10.2217/cns-2017-0026

**Published:** 2017-12-15

**Authors:** Hao-Wen Sim, Erin R Morgan, Warren P Mason

**Affiliations:** 1 Princess Margaret Cancer Centre, 610 University Avenue, Toronto, Ontario M5G 2M9, Canada

**Keywords:** bevacizumab, drug development, elderly, high-grade glioma, immunotherapy, molecular biology, tumor-treating fields

## Abstract

High-grade gliomas, including glioblastoma, are the most common malignant brain tumors in adults. Despite intensive efforts to develop new therapies for these diseases, treatment options remain limited and prognosis is poor. Recently, there have been important advances in our understanding of the molecular basis of glioma, leading to refinements in our diagnostic and management approach. There is new evidence to guide the treatment of elderly patients. A multitude of new agents have been investigated, including targeted therapies, immunotherapeutics and tumor-treating fields. This review summarizes the key findings from this research, and presents a perspective on future opportunities to advance the field.

**Practice points**

- Molecular markers now have an established role in the diagnosis and management of high-grade glioma, and have been incorporated into the updated WHO 5th Edition Classification of Tumors of the Central Nervous System.
- There is new evidence to guide management of patients over 65 years of age with high-grade glioma. Most of these patients will benefit from short-course radiotherapy with concurrent and adjuvant temozolomide.
- For unselected patients with high-grade glioma, the role of bevacizumab is limited.
- Tumor-treating fields have shown efficacy in patients with newly diagnosed glioblastoma. However, it does not meet established benchmarks for cost–effectiveness and has not been widely adopted as a therapy for patients with this disease.
- Immunotherapeutics are under evaluation for high-grade glioma. We await the forthcoming results from randomized Phase III trials.
- Many targeted therapies have been evaluated for high-grade glioma, but so far have lacked clinical utility. Efforts incorporating novel strategies are ongoing.

High-grade gliomas, including glioblastoma (GBM), anaplastic astrocytoma (AA) and anaplastic oligodendroglioma (AO), originate from the supporting neuroglial cells of the CNS. GBM, the most common and most aggressive of the primary brain tumors, typically presents in late adulthood. AA and AO affect a younger age group and generally have a more protracted clinical course. High-grade gliomas can be debilitating, owing to physical disability, cognitive impairment, personality change, depression and seizure disorder, and require complex multidisciplinary care.

For patients with newly diagnosed GBM, maximal safe resection followed by radiotherapy with concomitant and adjuvant temozolomide chemotherapy is the current standard of care. Median overall survival for patients with GBM remains poor and was 14.6 months in the landmark EORTC 26981/22981-NCIC CTG CE3 Phase III trial [1,2]. This same approach is used for patients with newly diagnosed AA, based on preliminary findings of the ‘Concurrent and Adjuvant Temozolomide in Non-1p/19q Deleted Anaplastic Glioma’ (CATNON) trial [3]. For patients with newly diagnosed AO, chemoradiotherapy with procarbazine, lomustine and vincristine (PCV) has demonstrable efficacy in Phase III trials [4,5]. However, due to the increased toxicity and treatment burden of PCV versus monthly temozolomide, many clinicians substitute temozolomide in this setting. Accordingly, a comparison of PCV against temozolomide is underway in the ‘Concomitant and Adjuvant Temozolomide Versus PCV Chemotherapy in Patients With Anaplastic Glioma or Low Grade Glioma’ (CODEL) trial (NCT00887146).

Tumor recurrence occurs in almost all patients. Options include repeat surgical debulking, radiotherapy, lomustine (CCNU), bevacizumab, etoposide and procarbazine. However, there is no global standard and prognosis is limited. Patients are encouraged to enroll in clinical trials to access novel therapies.

Clearly, there exists an unmet need for innovative approaches. In this review, we highlight important changes to the evidence base for high-grade glioma. We begin with a discussion of major updates, namely molecular neuropathology, the management of elderly patients, bevacizumab and tumor-treating fields (TTF). This is followed by an overview of clinical trials of immunotherapeutics and targeted therapies for high-grade gliomas. Finally, we offer our perspective on research priorities and speculate on the future directions of the field.

## Major updates

### Molecular biology of high-grade gliomas

There have been substantial advances in our understanding of the molecular aberrations found in malignant gliomas. Key discoveries include the isocitrate dehydrogenase (IDH) mutation, codeletion of the short arm of chromosome 1 and long arm of chromosome 19 (1p19q) and O^6^-methylguanine-DNA methyltransferase (MGMT) gene promoter methylation. These have emerged as being important determinants of treatment response and survival. Consequently, they are now routinely tested and have become fundamental to glioma classification.

IDH catalyzes the oxidative decarboxylation of isocitrate to α-ketoglutarate, and subsequently to the oncometabolite 2-hydroxyglutarate [6]. In turn, 2-hydroxyglutarate acts via a family of dioxygenases to impair epigenetic regulation and increase hypoxia-inducible factor 1-α. The prevalent *IDH1* R132H mutation is detectable with immunohistochemistry in over 90% of cases [7]. IDH mutations can also be identified by sequencing *IDH1* codon 132 and *IDH2* codon 172. These mutations are common in low-grade gliomas and secondary GBMs, and confer significantly improved prognosis [8].

The 1p19q codeletion is an unbalanced reciprocal translocation that is a characteristic of oligodendrogliomas. Multiple studies have demonstrated the favorable prognostic and predictive utility of the 1p/19q codeletion, although the biologic basis remains unclear. Specifically, in randomized Phase III trials evaluating chemoradiotherapy with PCV for AO, patients harboring the 1p19q codeletion derived greater benefit from PCV and lived substantially longer [4,5]. In contrast, partial 1p or 19q loss did not confer this significance.

MGMT gene promotor methylation causes epigenetic silencing of MGMT, which is necessary for DNA repair. Notably, based on review of randomized Phase III trials evaluating temozolomide in patients with GBM, those containing the MGMT gene promoter methylation obtained meaningful survival benefit from temozolomide, whereas those without the methylation did not [9]. Initially, MGMT status was assessed with immunohistochemistry and MGMT methylation-specific PCR; however, widespread clinical use was limited by numerous technical issues including poor reliability, reproducibility and the labor-intensive work [10,11]. Newer methods include bisulfite sequencing, pyrosequencing, high-resolution melt analysis and infinium methylation BeadChip, which have improved standardization and accuracy of MGMT testing [12,13].

In 2016, the WHO published the 5th Edition Classification of Tumors of the Central Nervous System [14]. This represents a seminal update, with the introduction of integrated diagnoses combining histology and molecular parameters for many entities. This incorporates the recently established prognostic and predictive information from IDH and 1p19q.

GBM is now subdivided into IDH wild-type (predominantly primary GBM, patients over 55 years of age, poor prognosis) and IDH-mutant entities (predominantly secondary GBM, younger patients, favorable prognosis). The diagnosis of AO requires IDH-mutant and 1p19q-codeleted status, whereas AA requires IDH-mutant and noncodeleted status. Importantly, both entities are IDH mutant; a glioma that is IDH wild-type with or without 1p19q codeletion instead represents a genomically unstable GBM. In addition, 1p19q codeletion is mutually exclusive with TP53 mutation and ATRX inactivation [15]. Accordingly, a glioma that is IDH-mutant, TP53-mutant and ATRX-inactivated is considered AA. Finally, the use of molecular parameters handles the problematic and indeterminate entity called anaplastic oligoastrocytoma, which was previously defined by a mixed histological pattern and was subject to poor interobserver agreement [16,17]. The combination of histology and molecular parameters effectively differentiates nearly all cases as either AO or AA. To facilitate clinical decision making, the current standard is to incorporate all the tissue-based information (histology, grade, molecular findings) into an integrated diagnosis, which is then reported to clinicians.

Molecular markers have significantly contributed to diagnostic precision in high-grade glioma, and yield important therapeutic implications. The next steps will be to improve understanding of clinical and molecular heterogeneity within glioma subtypes. Ongoing efforts include assessment of additional molecular markers, methylation profiling and a coordinated approach to histologic–molecular correlation as part of clinical trials.

### Treatment of high-grade gliomas in the elderly

Although GBM is predominantly a disease of older adults, with a median age of diagnosis of 64 and an increasing number of patients diagnosed over the age of 70 [18], management of this disease in the elderly remains a particular challenge. Compared with younger patients, those over the age of 65 have shorter overall survival [19–21]. The EORTC 26981/22981-NCIC CE3 study excluded patients over 70 [1,2], and although a subgroup analysis showed a trend toward benefit in patients over 60 years old, the degree of benefit diminished with increasing age [19]. Elderly patients are also more susceptible to toxicities of treatment, including radiation-induced cognitive deficits [22] and chemotherapy side effects [23]. GBM in elderly patients also seems to be biologically more aggressive, with a very low incidence of favorable prognostic markers such as IDH mutations and the glioma cytosine–phosphate–guanine island methylator phenotype, although MGMT status does not seem to vary with age [24].

This has led to efforts to tailor treatment to this patient population through the use of modified radiation schedules and selective use of temozolomide chemotherapy. The French ANOCEF study demonstrated a median survival benefit of 12 weeks with radiotherapy versus supportive care alone in elderly patients [25]. Subsequently, Roa *et al*. [26] demonstrated similar survival in patients over age 60 who received short-course radiotherapy (40 Gy in 15 fractions) versus the conventional regimen (60 Gy in 30 fractions). Patients treated with short-course radiotherapy were less likely to require increased corticosteroid doses after completing treatment. The Nordic study [27] randomized patients over age 60 with newly diagnosed GBM to standard radiotherapy (60 Gy in 30 fractions), hypofractionated radiotherapy (34 Gy in 10 fractions) or temozolomide monotherapy. This trial demonstrated longer overall survival (OS) in patients over the age of 70 treated with hypofractionated versus standard radiotherapy. In addition, this study demonstrated improved survival in MGMT-methylated patients treated with temozolomide, whereas MGMT status did not influence response to radiotherapy. The NOA-08 study [28] demonstrated that temozolomide monotherapy was noninferior to radiotherapy alone in elderly patients with MGMT-methylated GBM, while patients with unmethylated tumors showed improved survival with radiotherapy alone. In addition, for the frail elderly with Karnofsky performance score <70, Pérez-Larraya *et al*. [29] showed that temozolomide monotherapy was a well-tolerated regimen, and for the subgroup with MGMT-methylated GBM, survival outcomes were superior to historical control.

The role of combined chemoradiotherapy in elderly patients remained undefined until the recent publication of a Phase III trial, which randomized 562 elderly patients with GBM to either short-course radiotherapy alone (40 Gy in 15 fractions) or radiotherapy with concurrent temozolomide [30]. This trial demonstrated improved OS in patients treated with combined therapy (median 9.3 vs 7.6 months). Of note, survival benefit was greater in patients with MGMT-methylated tumors, although benefit was still observed in the unmethylated patient group.

Based on these studies, we can now propose a basic evidence-based approach for the first-line treatment of patients with GBM 65 years or older. For a minority of very fit, ‘physiologically young’ patients over 65, it remains reasonable to propose standard chemoradiotherapy, with a discussion of the possibility for increased toxicity in this age group. The majority of patients with adequate performance status and without comorbidities precluding combined therapy should receive short-course radiotherapy (40 Gy in 15 fractions) with concurrent and adjuvant temozolomide. Patients with poor performance status and the frail elderly may be offered best supportive care, or treatment based on MGMT status: temozolomide monotherapy for patients with MGMT-methylated GBM or short-course radiotherapy alone for unmethylated GBM.

### Role of bevacizumab

GBM is a highly vascular neoplasm, with abnormal vasculature characterized by tortuous blood vessels, vascular permeability and resulting hypoxia leading to the histological finding of pseudopalisading necrosis [31]. Tumor growth and invasion are intrinsically linked to hypoxia, which results in upregulation of hypoxia-inducible factor 1-α, and downstream upregulation of VEGF, which is associated with glioma cell stemness, mesenchymal phenotype and an immunosuppressive cellular milieu [32]. Thus, there is a strong biologic rationale for the use of antiangiogenic agents in GBM, and these drugs have thus been extensively studied as therapeutic targets in both newly diagnosed and recurrent GBM.

Bevacizumab, a humanized monoclonal antibody which binds VEGF-A, is the most extensively studied of the antiangiogenic agents for GBM. Bevacizumab was approved by the US FDA for use in recurrent GBM in 2009 [33]. The ‘Bevacizumab Alone and in Combination with Irinotecan in Recurrent GBM’ (BRAIN) study [34] was a randomized Phase II trial that assigned 167 patients with recurrent GBM to receive bevacizumab 10 mg/kg with or without irinotecan. This trial demonstrated objective response rates of 38 and 28% in patients treated with bevacizumab with and without irinotecan, respectively. Progression-free survival at 6 months (PFS-6) was 42% in patients treated with bevacizumab alone and 50% in the combination arm. In a single-arm study, 48 patients with recurrent GBM were treated with bevacizumab 10 mg/kg with irinotecan added upon disease progression, demonstrating an objective response rate of 35% and PFS-6 of 29% [35]. While these findings led to FDA approval for recurrent GBM in the USA, its use has not been approved in Europe due to concerns regarding the lack of a bevacizumab-free control arm, the modest improvement in OS and difficulties with interpreting MRI-based disease progression in patients treated with bevacizumab [36].

In the USA, widespread use of bevacizumab for recurrent GBM has limited the opportunity for further evaluation in this setting. In Europe, the randomized Phase II ‘Single-Agent Bevacizumab or Lomustine Versus a Combination of Bevacizumab Plus Lomustine in Patients with Recurrent GBM’ (BELOB) trial [37] showed promising results for the combination of bevacizumab and lomustine versus either agent alone. Unfortunately, these findings were not borne out in the subsequent Phase III trial which compared the combination of lomustine and bevacizumab with lomustine alone [38]. This trial showed no difference in OS, although there was a significant increase in PFS from 1.5 to 4.2 months in the combination arm. Several other Phase II trials have evaluated the combination of bevacizumab with a variety of other cytotoxic and targeted agents, including temozolomide, temsirolimus and erlotinib, but none have shown significant activity [31].

Similarly, bevacizumab has been tested in the setting of newly diagnosed GBM, with a series of Phase II trials using bevacizumab in combination with radiotherapy and temozolomide [39–41]. As seen in the recurrent setting, PFS was prolonged in comparison to historical controls (13–14 months), while the effect on OS was modest (10–21 months). Subsequently, two randomized Phase III trials were conducted, ‘A Study of Avastin in Combination With Temozolomide and Radiotherapy in Patients With Newly Diagnosed GBM’ (AVAGlio) [42] and RTOG-0825 [43]. These studies showed longer PFS in patients treated with bevacizumab, but failed to show OS benefit. Thus, despite encouraging preclinical results with *in vivo* activity and reduction of vasogenic edema, there is abundant high-quality evidence that bevacizumab is not indicated in unselected patients with newly diagnosed GBM.

### Role of alternating electric fields

The locoregional use of alternating electric fields to treat tumors, called TTF, represents a novel treatment modality [44]. Preclinical data showed that low-intensity (1–3 V/cm), intermediate-frequency (200 kHz) fields selectively disrupted microtubule assembly, thereby interfering with mitotic spindle formation and segregation of the two daughter cells during mitosis. This caused preferential apoptosis of rapidly dividing malignant cells. In contrast, lower frequencies caused undesirable depolarization of neurons and myocytes, and higher frequencies caused excess heat generation.

TTF is administered via transducer arrays, which are applied to the patient's shaved scalp for 18 h per day. The arrays are oriented to target the tumor volume based on contemporaneous imaging. The device is powered by a portable generator carried by the patient.

Two pivotal randomized Phase III trials have examined the efficacy of TTF in patients with GBM. The EF-11 trial [45] examined 237 patients with progressive GBM treated with either TTF or physician's choice of chemotherapy and failed to demonstrate improvement in PFS (median 2.2 vs 2.1 months; HR: 0.81; 95% CI: 0.60–1.09; p = 0.16) or OS (median 6.6 vs 6.0 months; HR: 0.86; 95% CI: 0.66–1.12; p = 0.27).

The second study, the EF-14 trial [46], investigated 695 patients with newly diagnosed GBM who had already completed temozolomide chemoradiotherapy. The patients were recruited from 83 institutions internationally and their characteristics were comparable to contemporary Phase III trials of newly diagnosed GBM. They received either TTF and temozolomide, or temozolomide alone for 6–12 cycles. The primary endpoint was PFS, which improved from a median of 4.0–7.1 months (HR: 0.62; 98.7% CI: 0.43–0.89; p = 0.001). In addition, OS in the intention-to-treat population improved from a median of 16.6–19.6 months (HR: 0.74; 95% CI 0.56–0.98; p = 0.03). This survival improvement was confirmed in preliminary reporting of the updated EF-14 results [47].

The predominant toxicities from TTF were local skin irritation and headache. Skin irritation was grade 1 or 2 in 43% of patients, and grade 3 in 2% of patients. Grade 1 or 2 headache was more frequently reported (TTF 21% vs control 14%). No additional systemic toxicity was identified with TTF. It was reported that quality of life was not adversely affected, measured by EORTC QLQ C-30 and BN20 questionnaires, performance status and mini-mental examination scores [48]. However, in our opinion, these measures may not fully encompass the decisions involved in patient decision making, such as the perceived stigmatization and inconvenience of wearing the TTF device. Compliance was only 75% in the EF-14 trial, which involved highly motivated patients.

There are other considerations when interpreting the EF-14 trial. First, patients and investigators were not blinded. Patients randomized to TTF received additional training and support necessary for use of the TTF device. Together, these may have conferred a placebo benefit. Another qualification was that randomization was performed after successful completion of chemoradiotherapy, such that the benefit of TTF may not be applicable to patients with primary refractory disease.

Of note, the financial cost of TTF relative to its efficacy poses a major impediment to implementation. A cost–effectiveness analysis of TTF using a decision-analysis Markov model was reported based on the EF-14 results and unit cost of €21,000 per month [49]. The incremental cost–effectiveness ratio was €549,909 per life-year gained (95% CI: 447,017–745,805). In Canada and many other countries, an intervention with incremental cost–effectiveness ratio >CAD$100,000 per life-year gained is typically viewed as poor resource utilization, although thresholds are frequently debated.

In summary, for newly diagnosed GBM, the EF-14 trial is supportive of TTF as being an efficacious treatment modality. However, the absolute benefit is modest and it does not meet established benchmarks for cost–effectiveness. For progressive GBM, there is insufficient evidence of benefit. The widespread adoption of TTF will therefore be contingent on identifying subgroups of patients with greatest benefit, and reduction in the market price of TTF to a commensurate level. Further studies are evaluating the utility of TTF for GBM, such as in combination with bevacizumab or stereotactic radiotherapy (NCT02663271, NCT02743078, NCT01894061, NCT01925573, NCT02343549).

## Novel therapies

### Immunotherapy in high-grade gliomas

The success of immune-based therapies in cancers such as melanoma and non-small-cell lung cancer (NSCLC), as well as other solid and hematologic malignancies, has led to a revolution in systemic cancer therapy. Clinical trials using these agents, which include immune checkpoint inhibitors, antitumor vaccines and autologous cell-based therapies, are ongoing for a wide variety of tumor types, including GBM.

While there is preclinical evidence to support activity of these agents in primary CNS malignancies, their use is complicated by both the unique immunologic milieu of the CNS and by the wide array of mechanisms used by high-grade gliomas to promote peritumoral immunosuppression and avoid immunologic surveillance. Within the CNS, access by activated T cells is limited by the glia limitans, a component of the blood–brain barrier [50], as well as the expression of Fas ligand, leading to apoptosis of Fas-expressing cytotoxic T-lymphocytes [51]. The tumor microenvironment of GBM is characterized by expression of immunosuppressive cytokines, such as TGF-β, IL-10, signal transducer and activator of transcription 3 and programmed-death ligand 1 [50–52]. In addition, systemic immune suppression is inherent in many patients with GBM, as evidenced by significant lymphopenia, and is likely exacerbated by lymphotoxic treatments such as radiation, temozolomide and corticosteroids [50,53]. For instance, treatment-induced lymphopenia has been associated with shorter OS in patients treated with standard chemoradiotherapy, and may also influence response to immune-based therapies [53]. It remains to be seen to what degree these factors will influence the efficacy of immunotherapy in primary CNS malignancies.

Antitumor vaccine therapy is one form of immunotherapy that has been extensively investigated in GBM. Broadly, these vaccines can be characterized as peptide- and cell-based vaccines, although there are many variations of each.

In peptide-based vaccination, patients are directly inoculated with one or more tumor-associated antigens in order to elicit an immune response against tumor cells. The peptide epitopes are selected based on immunogenicity, tumor specificity and homogeneity of expression. These proteins may be linked with carrier proteins to enhance immunogenicity, such as keyhole limpet hemocyanin, and are frequently administered with immune-stimulating adjuvants including granulocytic-macrophage colony-stimulating factor.

The most extensively evaluated vaccine-based therapy for glioma to date is rindopepimut, which consists of the amino acid sequence of EGFR variant III (EGFRvIII) conjugated to keyhole limpet hemocyanin. This primes dendritic cells against EGFRvIII, which then generates a specific immune response against GBM cells harboring the EGFRvIII mutation. Notably, this represents a combination of targeted and immunotherapeutic strategies. For patients with newly diagnosed GBM harboring the EGFRvIII mutation, trials evaluated the addition of rindopepimut to standard temozolomide chemoradiotherapy. In the Phase II ACT-III trial, median PFS and OS were 9.2 and 21.8 months, respectively, which were significantly longer than matched controls [54]. However, there was no survival benefit in the randomized Phase III ACT-IV trial when preliminary results were recently presented [55]. For patients with recurrent GBM harboring the EGFRvIII mutation, preliminary findings from the randomized Phase II ReACT trial showed significant OS improvement from 8.8 to 12.0 months with rindopepimut plus bevacizumab versus bevacizumab alone [56]. Of note, upon progression after treatment with rindopepimut, EGFRvIII expression was lost, although it is unknown whether this represents the natural evolution of disease or the results of immunologic selection.

Other vaccines targeting EGFRvIII are in early clinical testing, including ADU-623, which uses a live attenuated listeria vaccine targeting EGFRvIII in recurrent high-grade glioma (NCT01967758). Another target for peptide vaccines in glioma is IDH1, given that the *IDH1* R132H mutation is expressed in the majority of secondary GBM and is thought to represent a driver mutation in glioma development. Phase I studies are underway (NCT02454634, NCT02193347, NCT02771301).

In cell-based vaccination, autologous antigen-presenting cells (APCs) such as dendritic cells are harvested from the patient and primed with one or more tumor-associated antigens. Antigen loading may be performed with one or more peptide epitopes, or harvested APCs may be exposed to a lysate of the patient's own tumor cells in order to generate a personalized vaccine. The primed APCs are then reintroduced to the patient, resulting in antigen-specific T-cell activation. Other targets for cell-based vaccine therapy include tumor-derived mRNA, glioma stem cells and virus-derived antigens. While cell-based vaccines offer a unique opportunity for highly personalized therapy, and potentially limited toxicity due to the specificity of the targeted antigens, these agents are expensive and labor-intensive to produce. The process entails harvesting APCs through apheresis, culturing and differentiating the cells *in vitro*, priming through exposure to the desired antigen and preparation of the personalized vaccine. This presents challenges in terms of potentially unacceptable delays in treatment as well as cost–effectiveness concerns. To date, there are no reported Phase III trials of cell-based therapies, so clinical utility of these agents remains to be determined.

Immune checkpoint inhibition is another promising form of immunotherapy. There is preclinical evidence of their activity in murine models of glioma, as well as clinical responses in brain metastases of melanoma and NSCLC. Multiple clinical trials are underway using these agents both in newly diagnosed and recurrent GBM, and as monotherapy, dual checkpoint inhibition and combinations with other agents. Preliminary results of CheckMate-143, a randomized Phase III trial using nivolumab in combination with bevacizumab in patients with recurrent GBM, were recently reported and showed no benefit of the nivolumab with bevacizumab combination over bevacizumab alone [57]. So far, responses in early phase studies suggest that the activity of these agents in GBM may be modest at best, in comparison to the dramatic responses observed in melanoma and NSCLC [58]. This may be related in part to the relatively low mutational burden of malignant glioma and the underlying immunosuppressive cellular milieu. Nonetheless, we await the forthcoming trial results to inform our decision making.

### Targeted therapies

Targeted therapies represent a paradigm shift toward rational drug design. They selectively target signaling pathways, which are believed to be important for glioma growth and survival, based on our understanding of the fundamental processes driving malignancy [59]. The targeted therapy trials, which will be discussed, are summarized in Table 1.

**Table 1.** **Targeted therapies in high-grade gliomas.**

**Study**	**Year**	**Setting**	**N**	**Design**	**Findings**	**Ref.**
**EGF inhibition**						

Rich *et al*.	2004	Recurrent glioblastoma	57	Phase II trial Gefitinib 500–1000-mg daily	ORR: 0% PFS-6: 13%	[60]

Neyns *et al*.	2009	Recurrent glioblastoma or anaplastic glioma	55	Phase II trial Cetuximab 250 mg/m^2^ weekly	ORR: 6% PFS-6: 9%	[61]

van den Bent *et al*.	2009	Recurrent glioblastoma	110	Phase II trial 1. Erlotinib 150–200-mg daily 2. Temozolomide 150–200 mg/m^2^ D1–5 q4w or carmustine 60–80 mg/m^2^ D1–3 q8w	ORR: 4 vs 10% PFS-6: 11 vs 24%	[62]

Li *et al*.	2010	Newly diagnosed glioblastoma	192	Phase II trial Temozolomide chemoradiotherapy + mAb 425	mOS: 15.7 m	[63]

Peereboom *et al*.	2013	Recurrent glioblastoma	56	Phase II trial Erlotinib 150 mg daily + sorafenib 400 mg twice-daily	ORR: 5% PFS-6: 14%	[64]

Reardon *et al*.	2015	Recurrent glioblastoma	119	Phase II trial 1. Afatinib 40 mg daily + temozolomide 75 mg/m^2^ D1–21 q4w 2. Afatinib 40 mg daily 3. Temozolomide 75 mg/m^2^ D1–21 q4w	ORR: 14 vs 14 vs 21% PFS-6: 10 vs 3 vs 23%	[65]

Westphal *et al*.	2015	Newly diagnosed glioblastoma	149	Phase III trial 1. Temozolomide chemoradiotherapy + nimotuzumab 2. Temozolomide chemoradiotherapy	mPFS: 7.7 vs 5.8 m mOS: 22.3 vs 19.6 m	[66]

**EGF antibody drug conjugate**						

Gan *et al*. (abstract)	2014	Recurrent glioblastoma	12	Phase I trial ABT-414	ORR: 33%	[67]

Reardon *et al*.	2016	Newly diagnosed glioblastoma	45	Phase I trial Temozolomide chemoradiotherapy + ABT-414	mPFS: 6.1 m mOS: not reached	[68]

**EGF peptide vaccine**						

Schuster *et al*.	2015	Newly diagnosed glioblastoma expressing EGFRvIII	65	Phase II trial Temozolomide chemoradiotherapy + rindopepimut	mPFS: 9.2 m mOS: 21.8 m	[54]

Reardon *et al*. (abstract)	2015	Recurrent glioblastoma expressing EGFRvIII	72	Phase II trial 1. Rindopepimut + bevacizumab 2. Bevacizumab	ORR: 24 vs 17% PFS-6: 27 vs 11% mOS: 12.0 vs 8.8 m	[56]

Weller *et al*. (abstract)	2016	Newly diagnosed glioblastoma expressing EGFRvIII	745	Phase III trial 1. Temozolomide chemoradiotherapy + rindopepimut 2. Temozolomide chemoradiotherapy	Minimal residual disease mOS: 20.1 vs 20.0 m Nonminimal residual disease mOS: 14.8 vs 14.1 m	[55]

**VEGF inhibition**						

Batchelor *et al*.	2010	Recurrent glioblastoma	31	Phase II trial Cediranib 45-mg daily	ORR: 27% PFS-6: 26%	[69]

Iwamoto *et al*.	2010	Recurrent glioblastoma	35	Phase II trial Pazopanib 800-mg daily	ORR: 6% PFS-6: 3%	[70]

de Groot *et al*.	2011	Recurrent glioblastoma or anaplastic glioma	58	Phase II trial Aflibercept 4 mg/kg q2w	Glioblastoma ORR: 18% PFS-6: 8% Anaplastic glioma ORR: 44% PFS-6: 25%	[71]

Pan *et al*.	2012	Recurrent glioblastoma or anaplastic glioma	30	Phase II trial Sunitinib 50-mg daily D1–28 q6w	Glioblastoma ORR: 0% PFS-6: 17% Anaplastic glioma ORR: 0% PFS-6: 22%	[72]

Kreisl *et al*.	2012	Recurrent glioblastoma or anaplastic glioma	64	Phase II trial Vandetanib 300-mg daily	Glioblastoma ORR: 13% PFS-6: 7% Anaplastic glioma ORR: 7% PFS-6: 7%	[73]

Muhic *et al*.	2013	Recurrent glioblastoma	25	Phase II trial Nintedanib 200 mg twice-daily	ORR: 0% PFS-6: 4%	[74]

Reardon *et al*.	2013	Recurrent glioblastoma	41	Phase II trial Pazopanib 400-mg daily + lapatinib 1000-mg daily	PTEN/EGFRvIII positive PFS-6: 0% PTEN/EGFRvIII negative PFS-6: 15%	[75]

Kreisl *et al*.	2013	Recurrent glioblastoma	63	Phase II trial Sunitinib 37.5-mg daily	Bevacizumab naive PFS-6: 10% Bevacizumab resistant PFS-6: 0%	[76]

Batchelor *et al*.	2013	Recurrent glioblastoma	325	Phase III trial 1. Cediranib 20-mg daily + lomustine 110 mg/m^2^ q6w 2. Cediranib 30-mg daily 3. Placebo + lomustine 110 mg/m^2^ q6w	ORR: 17 vs 15 vs 9% PFS-6: 35 vs 25 vs 16 mPFS: 4.1 vs 3.0 vs 2.7 m mOS: 9.4 vs 8.0 vs 9.8 m	[77]

**PDGF inhibition**						

Lassman *et al*.	2011	Recurrent glioblastoma with overexpression of dasatinib molecular targets	50	Phase II trial Dasatinib 100 mg twice-daily	ORR: 0% PFS-6: 6%	[78]

**mTOR inhibition**						

Chang *et al*.	2005	Recurrent glioblastoma	43	Phase II trial Temsirolimus 250-mg intravenously weekly	ORR: 5% PFS-6: 2%	[79]

Galanis *et al*.	2005	Recurrent glioblastoma	65	Phase II trial Temsirolimus 250-mg intravenously weekly	ORR: 36% PFS-6: 8%	[80]

Kreisl *et al*.	2009	Recurrent glioblastoma	22	Phase II trial Everolimus 70-mg weekly + gefitinib 250-mg daily	ORR: 14% PFS-6: 0%	[81]

**PI3K inhibition**						

Pitz *et al*.	2015	Recurrent glioblastoma	33	Phase II trial Sonolisib 8-mg daily	ORR: 3% PFS-6: 17%	[82]

**PKC inhibition**						

Kreisl *et al*.	2010	Recurrent glioblastoma or anaplastic glioma	118	Phase II trial Enzastaurin 500–525-mg daily	Glioblastoma ORR: 30% PFS-6: 7% Anaplastic glioma ORR: 15% PFS-6: 16%	[83]

Wick *et al*.	2010	Recurrent glioblastoma	266	Phase III trial 1. Enzastaurin 500-mg daily q6w 2. Lomustine 100–130 mg/m^2^ q6w	ORR: 3 vs 4% PFS-6: 11 vs 19% mPFS: 1.5 vs 1.6 m mOS: 6.6 vs 7.1 m	[84]

**c-Met inhibition**						

Wen *et al*. (abstract)	2010	Recurrent glioblastoma	124	Phase II trial Cabozantinib 125–175-mg daily	PFS-6: 21%	[85]

Wen *et al*.	2011	Recurrent glioblastoma	61	Phase II trial Rilotumumab 10–20 mg/kg q2w	ORR: 0%	[86]

**Integrin inhibition**						

Reardon *et al*.	2008	Recurrent glioblastoma	81	Phase II trial 1. Cilengitide 500 mg twice-weekly 2. Cilengitide 2000 mg twice-weekly	ORR: 5 vs 13% PFS-6: 10 vs 15%	[87]

Gilbert *et al*.	2012	Recurrent glioblastoma undergoing surgery	30	Phase II trial Cilengitide 2000 mg twice-weekly	PFS-6: 12%	[88]

Stupp *et al*.	2014	Newly diagnosed glioblastoma with MGMT promoter methylation	545	Randomized Phase III trial 1. Temozolomide chemoradiotherapy + cilengitide 2000 mg twice-weekly 2. Temozolomide chemoradiotherapy	mPFS: 13.5 vs 10.7 m mOS: 26.3 vs 26.3 m	[89]

**Microtubule inhibition**						

Stupp *et al*.	2011	Recurrent glioblastoma	38	Phase II trial Sagopilone 16 mg/m^2^ q3w	ORR: 0% PFS-6: 7%	[90]

Chamberlain *et al*.	2014	Recurrent glioblastoma	56	Phase II trial Verubulin 3.3 mg/m^2^ D1, 8, 15 q4w	Bevacizumab naive PFS-6: 14% Bevacizumab resistant PFS-6: 8%	[91]

**Histone deacetylase inhibition**						

Galanis *et al*.	2009	Recurrent glioblastoma	66	Phase II trial Vorinostat 200 mg twice-daily D1–14 q3w	ORR: 3% PFS-6: 15%	[92]

Friday *et al*.	2012	Recurrent glioblastoma	37	Phase II trial Vorinostat 400-mg daily D1–14 q3w + bortezomib 1.3 mg/m^2^ D1, 4, 8, 11 q3w	ORR: 3% PFS-6: 0%	[93]

Lee *et al*.	2015	Recurrent glioblastoma or anaplastic glioma	39	Phase II trial Panobinostat 30 mg D1, 3, 5 q2w + bevacizumab 10 mg/kg q2w	Glioblastoma ORR: 29% PFS-6: 30% Anaplastic glioma ORR: 27% PFS-6: 47%	[94]

**TGF-β inhibition**						

Brandes *et al*.	2016	Recurrent glioblastoma	158	Phase II trial 1. Galunisertib 150 mg twice-daily D1–14 q4w + lomustine 100–130 mg/m^2^ q6w 2. Galunisertib 150 mg twice-daily D1–14 q4w 3. Placebo + lomustine 100–130 mg/m^2^ q6w	ORR: 1 vs 5 vs 0% PFS-6: 6 vs 15 vs 6% mPFS: 2 vs 2 vs 2 m mOS: 6.7 vs 8.0 vs 7.5 m	[95]

**XPO inhibition**						

Mau-Sørensen (abstract)	2016	Recurrent glioblastoma	35	Phase II trial 1. Selinexor 50 mg/m^2^ twice-daily ×3, then surgery 2. Selinexor 50 mg/m^2^ twice-daily 3. Selinexor 60 mg twice-daily 4. Selinexor 80-mg daily	Pooled results ORR: 11% PFS-6: 15%	[96]

D#: Day #; EGFRvIII: EGFR variant III; m: Month; mAb: Monoclonal antibody; MGMT: O6-methylguanine-DNA methyltransferase; mOS: Median overall survival; mPFS: Median progression-free survival; N: Number; ORR: Objective response rate; PFS-6: Progression-free survival rate at 6 months; PTEN: Phosphate and tensin homolog; q8w: Every # weeks; XPO: Exportin-1.

****The EGF receptor is overexpressed in many GBMs [97]. Early studies suggested that GBMs harboring a specific EGF receptor mutation (EGFRvIII) and intact phosphate and tensin homolog suppressor (PTEN) gene might respond favorably to EGF inhibition [98]. Numerous EGF receptor inhibitors were therefore evaluated in GBM trials, including the small molecule inhibitors gefitinib [60], erlotinib [62,64] and afatinib [65], and the monoclonal antibodies cetuximab [61], mAb 425 [63] and nimotuzumab [66]. Unfortunately, despite being a proven strategy in NSCLC and other malignancies, results have consistently showed limited activity in GBM, even among those with EGF receptor amplification.

A novel approach to EGF inhibition is the use of antibody–drug conjugates such as ABT-414. The antibody component of ABT-414 effectively binds to mutant EGF receptors despite the blood–brain barrier, then delivers the cytotoxic drug component directly to the tumor cell. This obviates the pharmacokinetic challenges with conventional EGF receptor inhibition. There have been encouraging results from preliminary studies of ABT-414 [67,68], and Phase III trials are now underway (NCT02573324, NCT02343406).

Another approach is the use of EGF peptide vaccines such as rindopepimut, and studies evaluating this vaccine in patients with GBM have been presented elsewhere in this review.

Since GBM is a highly vascular tumor, another group of targeted therapies have been developed against tumor angiogenesis. Important mediators of angiogenesis include VEGF, PDGF, mTOR, PI3K, PKC and hepatocyte growth factor (c-Met). Bevacizumab, a monoclonal antibody against VEGF, was discussed previously. Unfortunately, the vast armamentarium of other antiangiogenic agents targeting VEGF, PDGF, mTOR, PI3K, PKC and c-Met have uniformly failed to show an efficacy signal, and no reliable biomarkers have been identified to improve treatment selection [69–86].

Regarding novel drug targets, cilengitide is an integrin inhibitor, which mediates transmembrane interactions between cells and the surrounding stroma. Despite early suggestion of cilengitide activity in GBM with MGMT promoter methylation [87,88], there was no survival benefit in the larger randomized Phase III ‘Cilengitide Combined With Standard Treatment for Patients With Newly Diagnosed GBM With Methylated MGMT Promoter’ (CENTRIC) trial, so development was discontinued [89].

Sagopilone and verubulin target the microtubules, which are needed for cell division [90,91]. Vorinostat and panobinostat inhibit histone deacetylase, with the aim of reversing epigenetic changes associated with malignancy [92–94]. Galunisertib inhibits TGF-β receptor-1, which has been associated with several growth signaling pathways [95]. Selinexor is a selective inhibitor of exportin-1 (XPO), which causes accumulation of tumor suppressor proteins within the nucleus [96]. Discouragingly, all of these drugs lacked relevant activity when tested in patients with recurrent GBM.

Overall, targeted therapies have yet to demonstrate efficacy beyond standard cytotoxic chemotherapy. A consideration is whether adequate intratumoral drug concentrations are reached due to the blood–brain barrier, given presence of efflux transporters on the endothelial cells and limited passage of large-molecular-weight antibodies. The presence of intratumoral heterogeneity is another complicating factor, since only malignant cells bearing the relevant target will be affected. In addition, there are likely varying degrees of pathway redundancy, so that blockade of one pathway alone may be insufficient for cytotoxicity.

To further the development of effective targeted therapies, it is essential that the pharmacokinetic and pharmacodynamic properties of prospective drugs are fully characterized in GBM patients at an early stage. An example would be use of window-of-opportunity studies, whereby test doses of drug are administered preoperatively, then drug levels and proof of drug action can be evaluated in the resection specimen. It may be necessary for certain drugs to be administered via alternate routes such as direct intratumoral injection. Novel approaches will be required to circumvent intratumoral heterogeneity. Possibilities include drug conjugates or combinations with radiopharmaceuticals and immunotherapeutics, whereby we leverage bystander effect and the immune response to deal with adjacent tumor cells not bearing the relevant target. Finally, there is critical need for predictive biomarkers to select subgroups of patients to benefit from particular treatments. We anticipate the increased adoption of molecular sequencing of tumors, novel imaging such as magnetic resonance spectroscopy, and the development of radiomics-based imaging analysis will provide new opportunities for biomarker discovery.

## Conclusion & future perspective

Despite exhaustive efforts to investigate novel therapies, treatment options for high-grade glioma have not changed significantly in recent times. A limiting factor is that trials are often small and underpowered, or require lengthy periods of patient accrual and follow-up before meaningful results become available. Moving forward, it is essential that we take maximum advantage of our finite patient pool. First, we must renew our support of international, multicenter, collaborative trial efforts which offer the greatest opportunity for robust and generalizable information to inform clinical decision making. Second, we need to embrace innovative trial methodologies that will allow us to more rapidly and efficiently test novel therapies. An exemplar is the ‘GBM Adaptive Global Innovative Learning Environment’ (GBM AGILE), which will use an adaptive trial design founded on Bayesian statistics. Patients will be preferentially recruited to treatment arms showing signs of efficacy, whereas ineffective treatment arms will be rapidly discontinued and replaced by newer options. Biomarker testing will be integrated into the selection process. In so doing, we optimize our expenditure of time and resources to support novel therapies with the greatest potential.

To achieve the promise of highly personalized medicine, we need to build on our advances in molecular neuropathology. A commitment to biobank collection of surgical specimens will afford an essential and rich resource for this. We envisage that concerted efforts in genomic sequencing, proteomic analysis and methylation profiling of high-grade gliomas, coupled with advances in bioinformatics and machine learning to interpret this data, will provide the next important discoveries. Novel research should be encouraged, such as investigations into miRNAs, the tumor microenvironment, and the glymphatic and immune response. Beyond pathology, we anticipate integration of novel imaging biomarkers into the repertoire, driven by development of techniques such as magnetic resonance spectroscopy and radiomics-based imaging analysis.

Finally, we need to adapt new methods to counter the blood–brain barrier, tumor heterogeneity and treatment resistance. Innovative combinations of treatment modalities will present such opportunities. Neurosurgery provides valuable access to the peritumoral space. Targeted drug conjugates can selectively deliver cytotoxic chemotherapy, immunotherapeutics and radiopharmaceuticals directly to tumor cells. A vast suite of different immunotherapy approaches remain to be investigated. Sequencing of radiotherapy with immunotherapeutics can produce an abscopal effect, a concept which is being trialed in several solid tumor sites. All in all, treatments are limited only by our creative potential.

The future promises to be an era characterized by robust and efficient trial design, a resolute commitment to deciphering the key molecular neuropathology and an engagement with innovative combination therapy. Together, they offer a realistic prospect of ameliorating this devastating disease.
